# Dexmedetomidine preconditioning may attenuate myocardial ischemia/reperfusion injury by down-regulating the HMGB1-TLR4-MyD88-NF-кB signaling pathway

**DOI:** 10.1371/journal.pone.0172006

**Published:** 2017-02-21

**Authors:** Jing-jing Zhang, Ke Peng, Juan Zhang, Xiao-wen Meng, Fu-hai Ji

**Affiliations:** Department of Anesthesiology, First Affiliated Hospital of Soochow University, Suzhou, China; Indiana University School of Medicine, UNITED STATES

## Abstract

**Aims:**

To investigate whether dexmedetomidine (DEX) preconditioning could alleviate the inflammation caused by myocardial ischemia/reperfusion (I/R) injury by reducing HMGB1-TLR4-MyD88-NF-кB signaling.

**Methods:**

Seventy rats were randomly assigned into five groups: sham group, myocardial I/R group (I/R), DEX+I/R group (DEX), DEX+yohimbine+I/R group (DEX/YOH), and yohimbine+I/R group (YOH). Animals were subjected to 30 min of ischemia induced by occluding the left anterior descending artery followed by 120 min of reperfusion. Myocardial infarct size and histological scores were evaluated. The levels of IL-6 and TNF-α in serum and myocardium were quantified by enzyme-linked immunosorbent assay, and expression of HMGB1, TLR4, MyD88, IκB and NF-κB in the myocardial I/R area were determined with Western blot and immunocytochemistry.

**Results:**

Myocardial infarct sizes, histological scores, levels of circulating and myocardial IL-6 and TNF-α, the expression of HMGB1, TLR4, MyD88 and NF-κB, and the degradation of IκB were significantly increased in the I/R group compared with the sham group (P<0.01). DEX preconditioning significantly reduced the myocardial infarct size and histological scores (P<0.01 vs. I/R group). Similarly, the serum and myocardial levels of IL-6 and TNF-α, the expression of HMGB1, TLR4, MyD88 and NF-κB, and the degradation of IκB were significantly reduced in the DEX group (P<0.01 vs. I/R group). These effects were partly reversed by yohimbine, a selective α_2_-adrenergic receptor antagonist, while yohimbine alone had no significant effect on any of the above indicators.

**Conclusion:**

DEX preconditioning reduces myocardial I/R injury in part by attenuating inflammation, which may be attributed to the downregulation of the HMGB1-TLR4-MyD88-NF-кB signaling pathway mediated by the α_2_-adrenergic receptor activation.

## Introduction

In the ischemic heart, coronary reperfusion can effectively limit infarct size. Unfortunately, reperfusion itself can result in additional injury which is called “ischemia/reperfusion (I/R) injury”[1,2]. During reperfusion, a variety of cytokines such as tumor necrosis factor α (TNF-α) and interleukin 6 (IL-6) are released, triggering excessive regional inflammatory responses and provoking further myocardial damage. A number of studies have shown that inhibition of excessive inflammation reduced infarct size and ameliorated heart dysfunction induced by I/R injury [3–5].

Toll-like receptor 4 (TLR4) has been identified as a mediator of inflammation and organ injury in several I/R models including cerebral I/R, liver I/R and myocardial I/R [6–8]. During ischemia, a number of damage-associated molecule pattern (DAMP) proteins, such as S100B and high mobility group box-1 (HMGB1), are released from damaged tissues [9,10], and stimulate TLR4. Activation of TLR4 then promotes the activity of NF-κB through a MyD88-dependent pathway, which triggers the expressions of proinflammatory cytokines including IL-1, IL-6, and TNF-α, leading to further tissue damage [11].

As a DAMP protein, high mobility group box -1 (HMGB1) is generally known as a non-histone DNA binding protein, and is involved in stabilization of DNA and promotion of transcription. However, HMGB1 has recently been found to be passively released by necrotic cardiomyocytes in response to ischemia and acts as an early mediator of inflammation following I/R injury [9]. In addition, HMGB1 is also secreted actively by immune cells such as monocytes, macrophages and dendritic cells, which further enhances the inflammatory reaction [12]. More importantly, clinical data indicate that increased circulating HMGB1 levels are associated with cardiac and neurological outcomes, and even increased mortality, in cardiac disease patients [13–15]. Therefore, HMGB1 plays a critical role, through TLR4-mediated signaling, in cardiovascular diseases, and may represent an important therapeutic target.

Dexmedetomidine (DEX), a highly selective α_2_-adrenergic receptor agonist, is widely used as a sedative agent in clinical anesthesia, intensive care unit (ICU) management and pain treatment [16–18]. Our previous work [19] and other reports [20,21] have suggest that DEX administration improves cardiac outcomes during non-cardiac surgery. It was also reported that DEX preconditioning significantly reduces the incidence of reperfusion-induced ventricular arrhythmias and the infarct area in the animal myocardial I/R models [22–24]. In addition, more studies have shown that DEX exerts an anti-inflammatory effect by reducing the serum levels of inflammatory cytokines including IL-6 and TNF-α in both sterile and infectious inflammation models [25–27]. However, it remains unknown whether DEX confers cardioprotection by inhibiting inflammation in the myocardial I/R setting, and the underlying mechanisms have not been well explored. A recent study reported that DEX preconditioning attenuates the pulmonary inflammation caused by lung ischemia-reperfusion injury, in a rat model, by inhibiting the TLR4/MyD88/MAPK pathway [28]. Based upon these findings, we hypothesized that DEX preconditioning could alleviate the inflammation following myocardial I/R injury by reducing myocardial HMGB1 release and inhibiting the activity of the TLR4-MyD88-NF-κB signaling.

## Materials and methods

### Animals

Seventy adult male Sprague-Dawley rats weighing 250–300 g were obtained from Xinuosai Biological Technology Ltd. (Suzhou, China). All animals received standard diet and water, treated in accordance with the Guide for the Care and Use of Laboratory Animals published by the United States National Institute of Health (NIH, 8^th^ Edition, 2011). The study protocol was approved by the Soochow University Committee for the care and use of laboratory animals.

Euthanasia was planned if necessary at the end of a protocol as a means to relieve pain or distress that cannot be alleviated by analgesics, sedatives, or other treatments. Criteria for euthanasia included protocol-specific endpoints (such as degree of a physical or behavioral deficit or tumor size) that enabled a prompt decision by the veterinarian and the investigator to ensure that the endpoint was humane and, whenever possible, the scientific objective of the protocol was achieved [29,30]. Rats were intravenously administered with sodium pentobarbital (Sigma, St. Louis, USA, 120 mg/kg) for euthanasia by the right jugular vein. The dose was three times the anesthetic dose.

Ten animals died prior to the experimental endpoint, which was attributable to three possible reasons. Firstly, anesthesia was too deep for two animals at the beginning in sham group. Secondly, mechanical ventilation injured the lungs of three animals during the process of ischemia/reperfusion in I/R and DEX groups. Finally, during the procedure of surgical operation the heart function was severely compromised in five animals in DEX, DEX/YOH and YOH groups.

### Myocardial I/R model

All rats were subjected to myocardial I/R injury as previously described [31]. Briefly, rats were intraperitoneally administered with sodium pentobarbital (Sigma, St. Louis, USA, 40 mg/kg), and ventilated with a positive-pressure ventilator (ALC-V8, Shanghai, China). Adequacy of anesthesia was checked using a tail pinch test prior to the surgical procedure. The chest was opened at the fourth intercostal space, and the left anterior descending coronary artery (LAD) was ligated at the location of the 1–2 mm under the boundary of pulmonary conus and left auricle [32]. The LAD was occluded for 30 min (ischemia) and then released for 120 min (reperfusion). A heating pad was used to keep the body temperature at 37°C. A 24G intravenous catheter was cannulated into the right jugular vein for blood collecting and reagent treatments.

### Experimental protocols

Animals were randomly divided into 5 groups (n = 14/group): sham group (administration with normal saline 25 min before the sham surgery, i.e. the chest was opened but without LAD ligation); I/R group (administration with normal saline 25 min followed with LAD ligation and reperfusion); DEX group (DEX 6 μg/kg/h x 10 min + 0.7 μg/kg/h x 15 min followed by I/R); DEX/YOH group: yohimbine 12 mg/kg/h x 5 min + 0.5 mg/kg/h x 20 min, and five minutes later, DEX 6 μg/kg/h x 10 min + 0.7 μg/kg/h x 15 min followed by I/R; YOH group (yohimbine 12 mg/kg x 5 min + 0.5 mg/kg/h x 20 min followed by I/R). The dose of DEX was used according to that previously reported [33].

### Measurement of infarct and risk region sizes

The LAD was re-occluded at the end of the reperfusion and the heart was perfused with a 2% solution of Evans blue. Next, the left ventricle was harvested, with the atria and right ventricular tissues excised. The samples were serially sectioned from base to tip into six slices, approximately 2 mm each. The slices were then counterstained with 1% 2, 3, 5-triphenyltetrazolium chloride (Sigma Chemical) for 15–30 min at 37°C in phosphate buffer (pH 7.4) and immersed in 10% formaldehyde overnight. As a result of the Evans blue/TTC staining, the blue region of each slice indicates the normal myocardium while the non-blue region represents the ischemic myocardium which is called area at risk (AAR). Among the AAR, the white region represents the infarct area (IA). Images were captured using a digital camera (SZ-40, Olympus, Tokyo, Japan) and the AAR and IA were measured with Image J (NIH, USA). The infarct size was expressed as a percentage of the AAR.

### Histologic grading

The ventricles were harvested and fixed in 4% paraformaldehyde for 48 h and further prepared for paraffin sectioning. The sections were then stained with hematoxylin and eosin (HE). Histologic grading was carried out as previously described [34]. In brief, a score of 2.0 was given to those ventricles with florid and widespread leukocytic infiltration, and 1.0 given to those with definite but sparse lesions. Intermediate lesions were scored 1.5. Tissues with lesions less extensive than those graded 1.0 were given a score of 0.5. A score of 0 was given when no histologic abnormality was observed.

### Enzyme-Linked Immunosorbent Assay (ELISA)

Approximately 1.5~2 ml of blood was aspirated from the right jugular vein immediately after reperfusion for the measurement of the circulating levels of IL-6 and TNF-α. The serum was isolated after centrifugation at 3000×g for 15 min. After centrifugation, the serum was frozen at -80°C until enzyme-linked immunosorbent assay (ELISA) analysis was performed. The ventricles were rapidly harvested after reperfusion, immediately frozen in liquid nitrogen, and homogenized in RIPA buffer containing PMSF. The tissue homogenates were then centrifuged at 15,000x rpm for 30 min at 4°C. The supernatants were frozen at -80°C until ELISA analysis was performed. Levels of the IL-6 and TNF-α were quantified using specific ELISA kits for rats according to the manufacturer’s instructions (Multi Sciences Biotech Co., Ltd. Hangzhou, China). A standard curve for both cytokines was used to calculate the extracellular protein levels (pg/ml and pg/mg, respectively).

### Western blot analysis

The ventricles were rapidly harvested, immediately frozen in liquid nitrogen, and homogenized in RIPA buffer containing PMSF. The tissue homogenates were then centrifuged at 15,000x rpm for 30 min at 4°C. The supernatants were used for Western blots. In brief, 150 μg of protein lysates were separated in 10% SDS-PAGE and transferred onto a PVDF (Millipore Corp., Bedford, MA) membrane. Thereafter, the membranes were blocked with 5% nonfat milk for 2 h at room temperature and incubated with the primary antibodies against HMGB1 (1:1000, Abcam, USA), TLR-4 (1:200, Abcam, USA), MyD88 (1:200, Abcam, USA), IκB-α (1:10000, Abcam, USA) and NF-κB p-65 (1:500, Abcam, USA), respectively, at 4°C overnight. β-actin (1:1,000, Beyotime, Shanghai, China) was used as a loading control. The membranes were incubated for 2 h with HRP-conjugated secondary antibodies (1:1000, Beyotime, Shanghai, China). The specific protein bands were visualized by an ECL kit and then processed with the Image J for quantification.

### Immunohistochemistry analysis

For immunohistochemistry, the paraffin-embedded sections (5 μm) were rehydrated and microwaved for antigen retrieval with EDTA and pretreated with 0.3% H_2_O_2_. Subsequently, the sections were blocked with goat serum and were incubated overnight at 4°C in a humidified box with specific antibodies against TLR-4 (1: 200, Abcam, USA), MyD88 (1:200, Abcam, USA), P65 subunit of NF-κB (1: 500, Abcam, USA). Then, sections were incubated in a secondary antibody (1: 100, Zhongshan Biotechnology, China) solution for 2 h at room temperature. Finally, the sections were incubated in HRP-streptavidin (1:100, Zhongshan Biotechnology, China) for 30 min at 37°C, and the color reaction was developed with diaminobenzidine (DAB). For each section, five fields were randomly chosen from the surrounding infarction areas of the left ventricle and captured with a digital camera (SZ-40, Olympus, Tokyo, Japan).

### Statistical analysis

All data were expressed as mean ± standard error mean (SEM), and SPSS 17.0 statistical package was employed for statistical analysis. The size of risk and infarct areas, levels of IL-6 and TNF-α and the expressions of HMGB1, TLR-4, MyD88 and NF-κB were analyzed using one-way analysis of variance (ANOVA) followed by Tukey’s test. P < 0.05 was considered statistically significant.

## Results

### DEX preconditioning reduced I/R induced-myocardial infarct size

**Fig 1:**
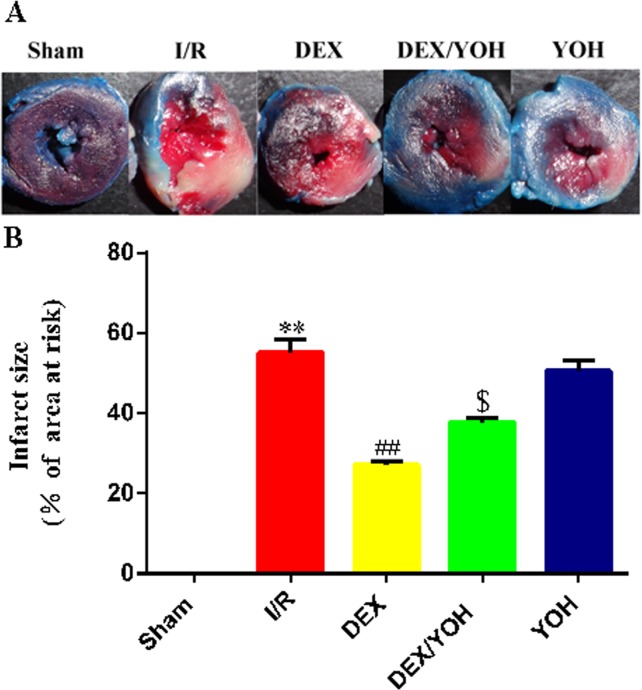
Dexmedetomidine preconditioning reduced I/R induced-myocardial infarct size. A. Representative images of myocardial infarction size in each group are shown. B. Quantitative analysis of myocardial infarct sizes (INF/AAR %) in each group. Values are shown as mean ± SEM. n = 4 per group. **P < 0.01, I/R vs. sham; ^##^ P < 0.01, DEX vs. I/R; ^$^ P<0.05, DEX/YOH vs. DEX.

We first explored whether DEX had any effects on I/R induced myocardial infarct size. As expected, I/R caused myocardial infarction compared with sham group (Fig 1A & 1B, **p<0.01, I/R vs. sham). However, DEX pretreatment significantly reduced the infarct size compared to that in I/R group (^##^p<0.01, DEX vs. I/R). Addition of yohimbine, the selective α_2_-adrenergic receptor antagonist, greatly attenuated the decrease in the infarct size achieved by DEX treatment (^$^p = 0.027, DEX/YOH vs. DEX), while yohimbine alone did not show any detectable impacts on the infarct size (p>0.05, YOH vs. I/R). Thus, we conclude that DEX preconditioning significantly shrinks the myocardial infarct size induced by I/R.

### DEX preconditioning attenuated I/R-induced myocardial structural injury

**Fig 2:**
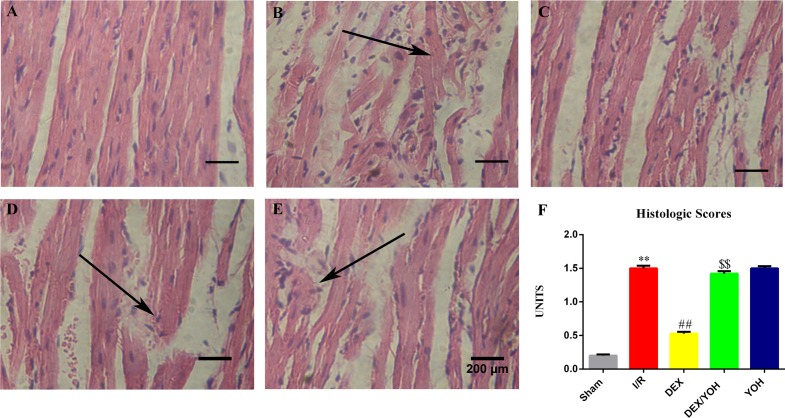
Dexmedetomidine preconditioning attenuated I/R-induced myocardial structural injury. H&E stained representative images of myocardial sections from sham (A), I/R(B), DEX (C), YOH/DEX (D), YOH (E) groups demonstrated histological changes. Magnification, ×400. Black arrows indicate the infiltrated inflammatory cells. F. Histologic scores from the hearts in each group. Bar = 200μm. Values are shown as mean ± SEM. n = 4 per group. **P < 0.01, I/R vs. sham group; ^##^P < 0.01, DEX vs. I/R; ^$ $^ P<0.01, DEX/YOH vs. DEX.

Next, we asked if DEX could alleviate the structural injury in the heart induced by I/R. While sham group showed normal myocardial structural organization (Fig 2A), severe histopathological alterations such as myocardial fiber disarrangement, extracellular edema, numerous leukocytes infiltration and an enlarged intercellular space were observed in the infarct areas of I/R group as anticipated, which led to a histological score of 1.51±0.074 (Fig 2B & 2F, **p<0.01, I/R vs. sham). Noticeably, DEX preconditioning significantly preserved more intact and less disrupted myocardial fibers compared to those in I/R group (Fig 2C), and had a histological score of 0.53±0.052 (Fig 2F, ^##^p<0.01, DEX vs. I/R). Co-treatment with DEX plus yohimbine significantly impaired the protective effects of DEX on the myocardial structure (Fig 2D), and increased the histological score to 1.42±0.073, the level equivalent to that of I/R group (Fig 2F, ^$ $^p<0.01, DEX/YOH vs. DEX). Yohimbine treatment alone did not affect the I/R-induced myocardial structural changes (Fig 2E &2 F). We therefore argue that DEX pretreatment ameliorates the I/R-linked myocardial structure damage, and that this protection is attenuated by yohimbine.

### DEX preconditioning attenuated the increase in the levels of circulating and myocardial IL-6 and TNF-α caused by I/R injury

**Fig 3:**
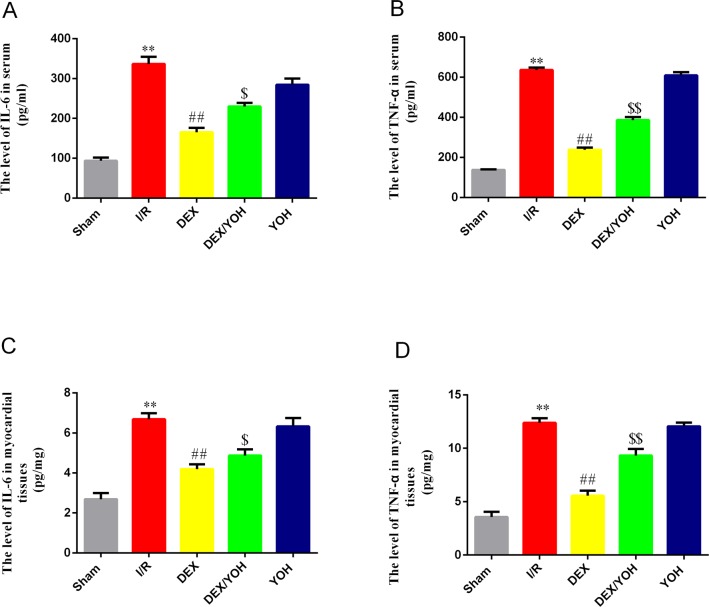
**Dexmedetomidine preconditioning attenuated the increase in the levels of circulating and myocardial IL-6 (A) and TNF-α (B) induced by I/R.** Values are shown as mean ± SEM, n = 5 per group for IL-6 and n = 4 per group for TNF-α. **P < 0.01, I/R vs. sham; ^##^P < 0.01, DEX vs. I/R; ^$^P<0.05, ^$ $^ P<0.01, DEX/YOH vs. DEX.

Given the above findings of cardiac protection offered by DEX pretreatment against I/R injury, we next asked if DEX could reduce the circulating and myocardial levels of pro-inflammatory cytokines in the I/R rats. Compared with sham group, I/R significantly increased the serum and myocardial levels of IL-6 and TNF-α (Fig 3A–3D, **p<0.01, vs. sham). However, DEX pretreatment remarkably decreased both the serum and myocardial levels of IL-6 and TNF-α (^##^p<0.01, DEX vs. I/R), while yohimbine partly reversed this effect (^$^p<0.05, ^$ $^p<0.01, DEX/YOH vs. DEX). Thus, consistent with the above findings, DEX treatment also inhibits the increase in the levels of circulating and myocardial IL-6 and TNF-α, and this inhibitor effect is attenuated by co-treatment with yohimbine.

### DEX preconditioning inhibited the HMGB1-TLR-4-MyD88-NF-κB signaling pathway

**Fig 4:**
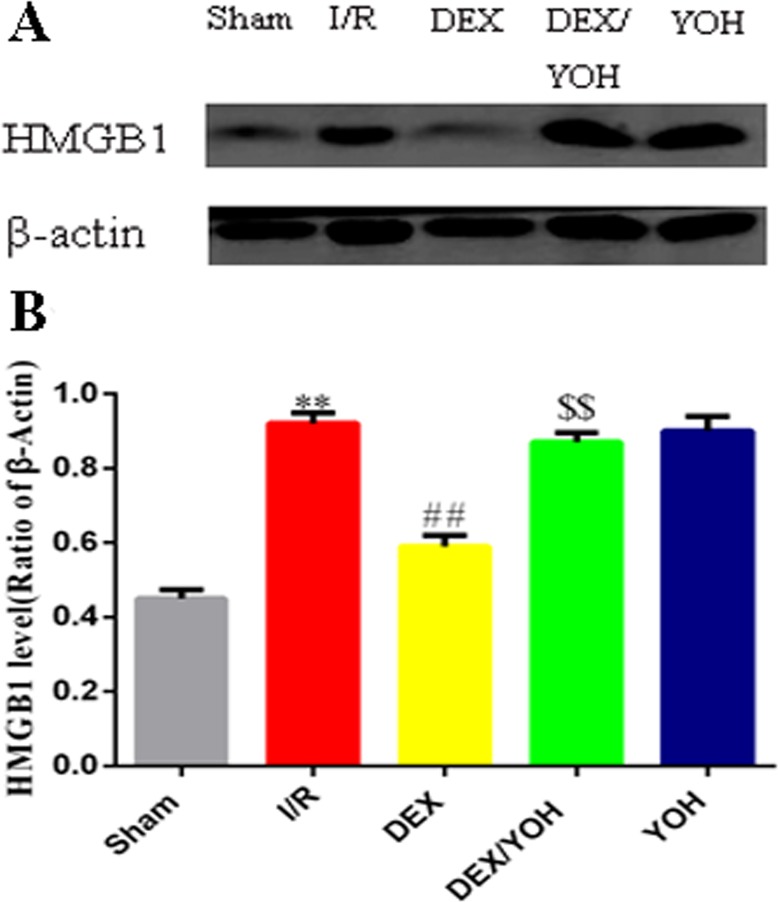
Dexmedetomidine preconditioning attenuated the increase in the expression of HMGB1 in the I/R-injured myocardium. A. Western blots showed the levels of HMGB1 in the hearts from the mice of each group. β-actin served as a loading control. B. Quantification of A. Values are shown as mean ± SEM. n = 4 per group. **P<0.01, I/R vs. sham; ^##^P<0.01, DEX vs. I/R; ^$ $^P<0.01, YOH/DEX vs. DEX.

**Fig 5:**
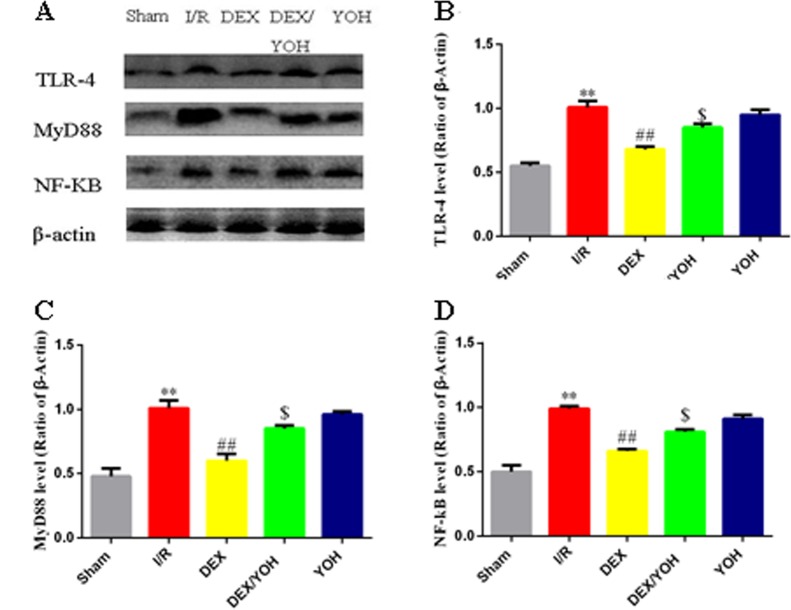
Dexmedetomidine preconditioning attenuated the increase in the expressions of TLR-4, MyD88 and NF-κB in the I/R-injured myocardium as revealed by Western blot. A. Western blots showed the levels of TLR-4, MyD88 and NF-κB in the hearts of mice from each group. β-actin served as a loading control. B, C and D. Quantifications of the TLR-4, MyD88 and NF-κB expressions, respectively, in each group. Values are shown as mean ± SEM. n = 4 per group. **P<0.01, I/R vs. sham group; ^##^P<0.01, DEX vs. I/R; ^$^P<0.05, YOH/DEX vs. DEX.

**Fig 6:**
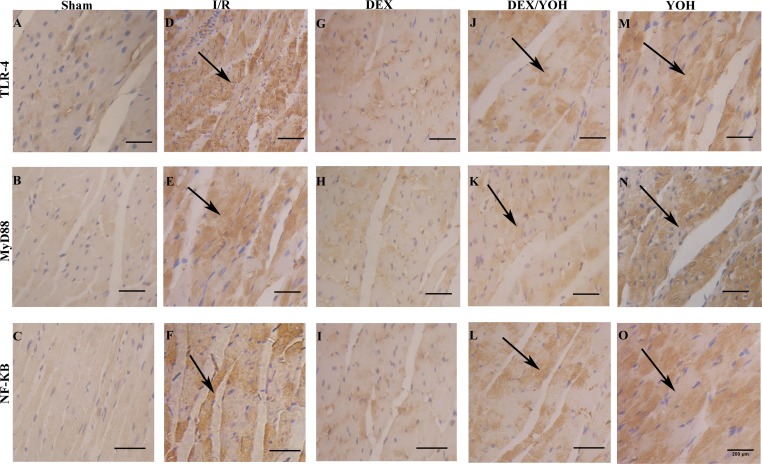
Dexmedetomidine preconditioning attenuated the increase in the expressions of TLR-4, MyD88 and NF-κB in the I/R-injured myocardium as revealed by immunohistochemistry. Representative images of immunostaining for TLR-4 (A, D, G, J, M), MyD88 (B, E, H, K, N) and NF-κB (C, F, I, L, O) in the sections of mouse hearts from different groups are shown. Black arrows indicate the positive staining of TLR-4, MyD88 or NF-κB, respectively. Bar scale: 200 μm. n = 4 per group.

**Fig 7:**
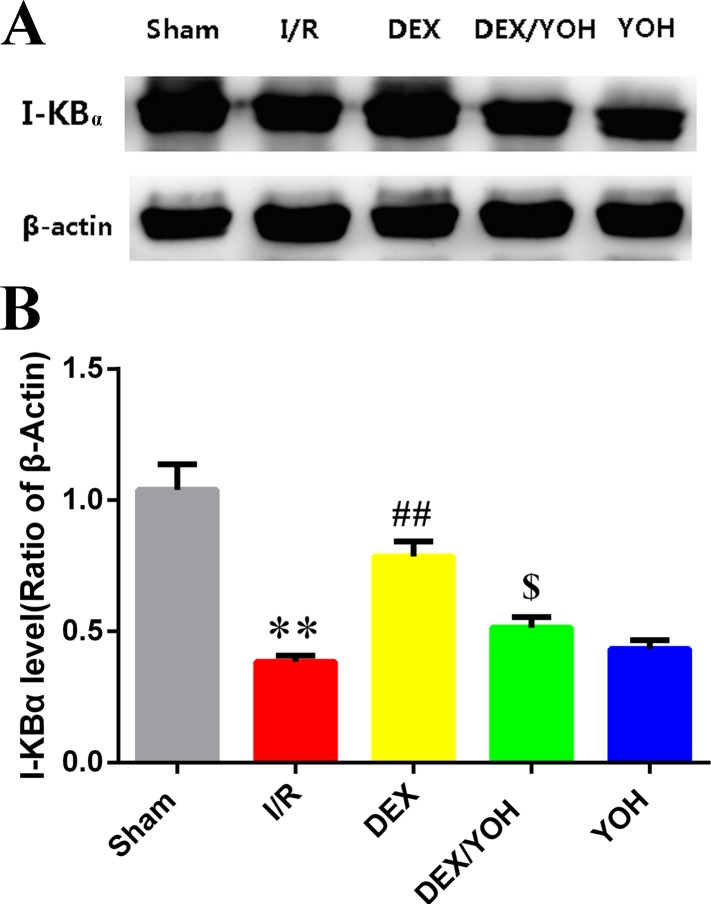
Dexmedetomidine preconditioning increased the expressions of IκB-α in the I/R-injured myocardium. A. Western blots showed the levels of IκB-α in the hearts of mice from each group. β-actin served as a loading control. B. Quantification of the IκB-α expression, respectively, in each group. Values are shown as mean ± SEM. n = 4 per group. **P<0.01, I/R vs. sham group; ^##^P<0.01, DEX vs. I/R; ^$^P<0.05, YOH/DEX vs. DEX.

Based on the above results, we found that DEX preconditioning significantly prevented the myocardium from the insult caused by I/R injury. This protection could be associated with its anti-inflammatory effect because a significant reduction of IL-6 and TNF-α levels in the serum was observed. To explore the underlying mechanism(s), we examined the effects of DEX on a signaling pathway involving HMGB1, a crucial mediator in the pathophysiology of the inflammatory response that occurs during the I/R-induced myocardial injury. Consistent with a previous study [9], we observed that I/R injury increased the level of HMGB1 in myocardium (Fig 4A & 4B, **p<0.01, I/R vs. sham), which was significantly reduced by DEX pretreatment (^##^p<0.01, DEX vs. I/R). Yohimbine largely abolished the effect of DEX (^$ $^p<0.01, DEX/YOH vs. DEX). Since the TLR4-MyD8-NF-κB signaling was shown to sit downstream of HMGB1 [33], we examined the changes in the expression of these three factors in the myocardium during I/R as well as treatments by DEX and yohimbine. As shown in Fig 5A–5D, the levels of these three factors, TLR4, MyD8, and NF-κB, were significantly elevated in the injured myocardium by I/R (**p<0.01, I/R vs. sham), and this elevation was greatly diminished by DEX treatment (^##^p<0.01, DEX vs. I/R). However, co-treatment of yohimbine partly abolished the inhibitory effects of DEX on the increase in the levels of TLR4, MyD8, and NF-κB (^$^p<0.05, DEX/YOH vs. DEX). The aforementioned changes of the levels of TLR4, MyD8, and NF-κB in the I/R-induced myocardium and treatment were further supported by the histological examination as shown in Fig 6. The expressions of TLR-4, MyD88 and NF-κB were increased in I/R (Fig 6D, 6E and 6F) and YOH (Fig 6M, 6N and 6O) groups, compared with those in sham group (Fig 6A, 6B and 6C), which were remarkably reduced in DEX group (Fig 6G, 6H and 6I). The inhibition of the increase in the levels of these factors by DEX was suppressed by co-treatment with yohimbine (Fig 6J, 6K and 6L). The expression of IκB-α was significantly decreased in I/R and YOH groups, compared with those in sham group, which was increased in DEX group. The increase in the levels of IκB-α by DEX was suppressed by co-treatment with yohimbine (Fig 7). Collectively, these findings suggest that DEX protects I/R-induced myocardial injury at least in part through targeting the HMGB1TLR4-MyD8-NF-κB signaling.

## Discussion

The current study offers new insights into the anti-inflammatory effect of DEX, and provides the first evidence, to our best knowledge, of the link between DEX and HMGB1 and its downstream signaling pathway in the myocardial I/R setting. We observed that DEX exerted a similar cardioprotective effect against I/R injury as has been previously reported [23], reducing infarct size and decreasing levels of circulating TNF-α and IL-6. Moreover, DEX preconditioning decreased expression of HMGB1, TLR-4, MyD88 and NF-κB in the ischemic-reperfused myocadium. We also observed that these benefits offered by DEX were blocked by yohimbine, an α_2_-adrenergic receptor antagonist. Taken together, these findings support the premise that DEX preconditioning attenuates the I/R-induced inflammatory response in the myocardium by reducing the cardiac HMGB1 levels and inhibiting its downstream TLR4-MyD88-NF-κB signaling.

Activation of the innate immune system following sterile injury mainly occurs when “danger signals” released from the damaged cells (so called DAMPs) interact with pattern recognition receptors (PRRs). In the myocardial I/R setting, danger signals including HMGB1 and Heat shock proteins (HSPs) are released from the necrotic myocardium and bind to their respective receptors such as TLR4, thus initiating the downstream MyD88-NF-кB signaling cascade [35,36]. Previous studies using either knockout of TLR4 [37] or administration of the TLR4 antagonist [38] have revealed significant decreases in NF-кB expression and corresponding inflammatory cell infiltration, pointing to the potential role for TLR4 in mediating the inflammatory reaction and suggesting that a timely interception of TLR4 might be critical to prevent adverse myocardial insult. In its inactive state, the NF-κB dimer is present in the cytosol, where it binds to an inhibitory protein, IκB. Activation of NF-κB by several stimuli induces the release and degradation of the inhibitory protein IκB from the dimeric complex, followed by phosphorylation of NF-κB p65 and translocation to the nucleus [39,40]. In our study, myocardial I/R induced parallel increases in the expression levels of TLR4 and HMGB1 (one of the ligands for TLR4) as well as the downstream effectors including MyD88 and NF-кB. The activation of NF-κB was further evidenced by the degradation of IκB. The changes observed in this signaling pathway were accompanied by the enlarged infarct size and heightened histologic damage. However, preconditioning with DEX efficiently alleviated all these I/R-linked pathological changes, which were coincident with the inhibition of the expressions of HMGB1, TLR4, MyD88, and NF-кB in the injured myocardium. Therefore, it is highly likely that DEX protected the myocardium from I/R through suppressing the HMGB1-TLR4-MyD88-NF-кB signaling, at least partially if not fully.

Previously, DEX was reported to have the capacity to inhibit the overproduction of inflammatory cytokines such as TNF-α and IL-6 in several acute inflammatory animal models [41–43]. Further studies have shown that DEX was associated with the stimulation of the α_2_-receptors in the central nervous system, which leads to the reduction of the sympathetic tone and shifts the balance towards the benefit of vagal nerve [44]. In addition, Kawada et al recently reported that intravenous DEX administration significantly enhanced the central vagal tone as evidenced by increased myocardial interstitial ACh levels, which was inhibited by the activation of the sympathetic nerve [45]. Given that 1) the vagal anti-inflammatory pathway was identified as a novel inhibitory mediator for the systematic and regional inflammation [35]; 2) HMGB1 was shown to play a pivotal role in the early stage of I/R event and aggravate cardiac injury [9]; 3) activation of the vagal nerve by acetylcholine or electroacupuncture significantly reduced HMGB1 release and inflammation [46]; and 4) we observed a significant reduction in the I/R-induced cardiac HMGB1 release and downstream inflammation in the DEX-treated mouse hearts, we therefore reckon that the downregulation of HMGB1 and its downstream signaling pathway by DEX might be a functional outcome of the stimulation of the cholinergic anti-inflammatory pathway. Future experiments will be needed to verify this premise.

There were also some limitations in the present study. First, we found that the anti-inflammatory effect of DEX was abolished by yohimbine, indicating that DEX induced anti-inflamatory effect mainly via the activation of α_2_-adrenoceptors. α_2_-Adrenoceptors were first discovered in the presynaps of the adreno-neurons in the central and peripheral nervous systems [47]. However, a recent in vitro study on the human kidney proximal tubular (HK2) cells by Gu et al. [41] has shown that DEX provides a renoprotective effect, which can be reversed by atipamezole, another selective α2-adrenoceptor antagonist, suggesting that α2-adrenoceptors may also be present in some non-neuronal cell types of the body. Therefore, we cannot exclude the possibility that a2-adrenoceptors are also present in the parenchymal cells in the myocardium (such as cardiomyocytes and fibroblasts), through which DEX may play its ant-inflammatory role. Second, HMGB1 has been found to interact with TLR2 and those receptors for advanced glycation end products (RAGE). However, RAGE only plays a minor role on the HMGB1-mediated macrophage activation in the reperfusion-induced tissue injury [48]. Besides, HMGB1 was shown to rapidly bind to TLR-2 and inhibit its expression, subsequently abolishing the HMGB1-induced NF-кB activation [49]. Therefore, whether TLR2 and RAGE are involved in DEX-induced inhibition of the HMGB1 release warrants further exploration.

In summary, we demonstrate that DEX preconditioning protects the hearts from I/R induced injury in a rat model. Mechanistically, DEX reduces the levels of circulating proinflammatory cytokines IL-6 and TNF-α at least partially by inhibiting the HMGB1-TLR4-MyD88-NF-κB signaling pathway as a result of α_2_-adrenergic receptor stimulation. These findings support the notion that DEX is not only an available sedative, but also plays an important role in the peri-operative cardioprotection. Hence, cardiovascular patients may benefit from DEX treatment during the perioperative period.

## Conclusions

DEX preconditioning protects the hearts from I/R induced injury in a rat model. Mechanistically, DEX reduces the levels of circulating proinflammatory cytokines IL-6 and TNF-α at least partially by inhibiting the HMGB1-TLR4-MyD88-NF-κB signaling pathway as a result of α_2_-adrenergic receptor stimulation.

## Supporting information

S1 File Original data. Table A in S1 File: infarct sizes. Table B in S1 File: histologic grading. Table C in S1 File: ELISA result of IL-6 in serum. Table D in S1 File: ELISA result of TNF-α in serum. Table E in S1 File: ELISA result of IL-6 in myocardial tissues. Table F in S1 File: ELISA result of TNF-α in myocardial tissues. Table G in S1 File: Western blot result of HMGB1 in myocardium. Table H in S1 File: Western blot result of TLR-4 in myocardium. Table I in S1 File: Western blot result of MyD88 in myocardium. Table J in S1 File: Western blot result of NF-ĸB in the myocardium. Table K in S1 File: Western blot result of IκB-α in the myocardium.
(DOC)
