# Supplementary material for: Dexmedetomidine preconditioning may attenuate myocardial ischemia/reperfusion injury by down-regulating the HMGB1-TLR4-MyD88-NF-кB signaling pathway
Source: PLoS One. 2017 Feb 21;12(2):e0172006. doi: 10.1371/journal.pone.0172006 (PMC5319750; doi:10.1371/journal.pone.0172006)
Supplement: S1 File — Table A in S1 File: infarct sizes. Table B in S1 File: histologic grading. Table C in S1 File: ELISA result of IL-6 in serum. Table D in S1 File: ELISA result of TNF-α in serum. Table E in S1 File: ELISA result of IL-6 in myocardial tissues. Table F in S1 File: ELISA result of TNF-α in myocardial tissues. Table G in S1 File: Western blot result of HMGB1 in myocardium. Table H in S1 File: Western blot result of TLR-4 in myocardium. Table I in S1 File: Western blot result of MyD88 in myocardium. Table J in S1 File: Western blot result of NF-ĸB in the myocardium. Table K in S1 File: Western blot result of IκB-α in the myocardium. (DOC) [file pone.0172006.s001.doc]

**Supporting Information 1. Original data**

Table A in S1 file: infarct sizes

| Group (n=4) | 1 | 2 | 3 | 4 | mean$\pm$SEM |
| --- | --- | --- | --- | --- | --- |
| Sham | 0 | 0 | 0 | 0 | 0 |
| I/R | 50 | 58 | 49 | 63 | 55$\pm$3.34^**^ |
| DEX | 25 | 30 | 27 | 26 | 27$\pm$1.08^##^ |
| DEX/YOH | 40 | 35 | 39 | 37 | 37.751.11^$^ |
| YOH | 52 | 44 | 57 | 49 | 50.5$\pm$2.72 |

Table B in S1 file: histologic grading

| Group (n=4) | 1 | 2 | 3 | 4 | mean$\pm$SEM |
| --- | --- | --- | --- | --- | --- |
| Sham | 0.21 | 0.17 | 0.25 | 0.18 | 0.2$\pm$0.02 |
| I/R | 1.58 | 1.43 | 1.45 | 1.55 | 1.5$\pm$0.04^**^ |
| DEX | 0.47 | 0.55 | 0.59 | 0.51 | 0.53$\pm$0.03^##^ |
| DEX/YOH | 1.35 | 1.40 | 1.39 | 1.52 | 1.420.04^$$^ |
| YOH | 1.53 | 1.48 | 1.42 | 1.56 | 1.50$\pm$0.03 |

Table C in S1 file: ELISA result of IL-6 in serum

| Group (n=5) | 1 | 2 | 3 | 4 | 5 | mean$\pm$SEM |
| --- | --- | --- | --- | --- | --- | --- |
| Sham | 106.67 | 87.13 | 118.04 | 78.95 | 79.01 | 93.96$\pm$7.86 |
| I/R | 376.34 | 385.56 | 316.07 | 302.69 | 301.24 | 336.38$\pm$18.44^**^ |
| DEX | 178.90 | 135.44 | 146.14 | 187.05 | 180.64 | 165.63$\pm$10.37^##^ |
| DEX/YOH | 216.15 | 220.34 | 210.62 | 249.63 | 254.06 | 230.169.01^$^ |
| YOH | 312.24 | 307.09 | 226.76 | 290.62 | 286.16 | 284.57$\pm$15.25 |

Table D in S1 file: ELISA result of TNF-α in serum

| Group (n=4) | 1 | 2 | 3 | 4 | mean$\pm$SEM |
| --- | --- | --- | --- | --- | --- |
| Sham | 140.82 | 143.92 | 134.64 | 131.54 | 137.73$\pm$2.82 |
| I/R | 637.35 | 670.97 | 620.61 | 615.59 | 636.13$\pm$12.51^**^ |
| DEX | 267.56 | 234.44 | 229.72 | 221.87 | 238.4$\pm$10.1^##^ |
| DEX/YOH | 401.88 | 414.83 | 385.74 | 342.35 | 386.215.8^$$^ |
| YOH | 602.24 | 613.92 | 568.97 | 650.78 | 608.98$\pm$16.88 |

Table E in S1 file: ELISA result of IL-6 in myocardial tissues

| Group (n=5) | 1 | 2 | 3 | 4 | 5 | mean$\pm$SEM |
| --- | --- | --- | --- | --- | --- | --- |
| Sham | 3.13 | 2.71 | 2.36 | 2.43 | 2.80 | 2.69$\pm$0.14 |
| I/R | 6.93 | 6.72 | 6.32 | 6.44 | 7.02 | 6.69$\pm$0.14^**^ |
| DEX | 3.90 | 4.31 | 4.52 | 4.22 | 4.07 | 4.20$\pm$0.11^##^ |
| DEX/YOH | 4.72 | 4.41 | 5.21 | 4.99 | 5.04 | 4.870.14^$^ |
| YOH | 6.24 | 6.14 | 6.53 | 5.81 | 6.92 | 6.33$\pm$0.19 |

Table F in S1 file: ELISA result of TNF-α in myocardial tissues

| Group (n=4) | 1 | 2 | 3 | 4 | mean$\pm$SEM |
| --- | --- | --- | --- | --- | --- |
| Sham | 4.18 | 3.07 | 3.69 | 3.31 | 3.56$\pm$0.24 |
| I/R | 12.15 | 12.98 | 12.41 | 11.99 | 12.38$\pm$0.22^**^ |
| DEX | 5.35 | 6.09 | 4.99 | 5.80 | 5.56$\pm$0.24^##^ |
| DEX/YOH | 8.64 | 9.10 | 10.11 | 9.45 | 9.320.31^$$^ |
| YOH | 12.04 | 12.28 | 11.58 | 12.32 | 12.05$\pm$0.17 |

Table G in S1 file: Western blot result of HMGB1 in myocardium

| Group (n=4) | 1 | 2 | 3 | 4 | mean$\pm$SEM |
| --- | --- | --- | --- | --- | --- |
| Sham | 0.45 | 0.38 | 0.46 | 0.50 | 0.45$\pm$0.02 |
| I/R | 0.95 | 0.85 | 0.89 | 0.98 | 0.92$\pm$0.03^**^ |
| DEX | 0.57 | 0.61 | 0.65 | 0.51 | 0.59$\pm$0.03^##^ |
| DEX/YOH | 0.89 | 0.92 | 0.87 | 0.80 | 0.870.03^$$^ |
| YOH | 0.81 | 0.99 | 0.93 | 0.87 | 0.9$\pm$0.04 |

Table H in S1 file: Western blot result of TLR-4 in myocardium

| Group (n=4) | 1 | 2 | 3 | 4 | mean$\pm$SEM |
| --- | --- | --- | --- | --- | --- |
| Sham | 0.55 | 0.61 | 0.49 | 0.56 | 0.55$\pm$0.02 |
| I/R | 1.02 | 0.95 | 0.93 | 1.15 | 1.01$\pm$0.05^**^ |
| DEX | 0.69 | 0.68 | 0.62 | 0.73 | 0.68$\pm$0.02^##^ |
| DEX/YOH | 0.88 | 0.91 | 0.82 | 0.77 | 0.850.03^$^ |
| YOH | 0.99 | 0.89 | 0.87 | 1.04 | 0.95$\pm$0.04 |

Table I in S1 file: Western blot result of MyD88 in myocardium

| Group (n=4) | 1 | 2 | 3 | 4 | mean$\pm$SEM |
| --- | --- | --- | --- | --- | --- |
| Sham | 0.32 | 0.48 | 0.61 | 0.53 | 0.48$\pm$0.06 |
| I/R | 0.88 | 0.97 | 1.04 | 1.16 | 1.01$\pm$0.06^**^ |
| DEX | 0.45 | 0.59 | 0.69 | 0.67 | 0.60$\pm$0.05^##^ |
| DEX/YOH | 0.82 | 0.80 | 0.91 | 0.87 | 0.850.02^$^ |
| YOH | 0.89 | 0.96 | 1.01 | 0.98 | 0.96$\pm$0.03 |

Table J in S1 file: Western blot result of NF-ĸB in the myocardium

| Group (n=4) | 1 | 2 | 3 | 4 | mean$\pm$SEM |
| --- | --- | --- | --- | --- | --- |
| Sham | 0.37 | 0.61 | 0.47 | 0.53 | 0.5$\pm$0.05 |
| I/R | 0.96 | 1.04 | 0.95 | 0.99 | 0.99$\pm$0.02^**^ |
| DEX | 0.63 | 0.71 | 0.67 | 0.64 | 0.66$\pm$0.02^##^ |
| DEX/YOH | 0.83 | 0.79 | 0.85 | 0.75 | 0.810.02^$^ |
| YOH | 0.84 | 0.99 | 0.94 | 0.88 | 0.91$\pm$0.03 |

Table K in S1 file: Western blot result of IκB-α in the myocardium

| Group (n=4) | 1 | 2 | 3 | 4 | mean$\pm$SEM |
| --- | --- | --- | --- | --- | --- |
| Sham | 1.26 | 0.84 | 1.14 | 0.92 | 1.04$\pm$0.10 |
| I/R | 0.37 | 0.40 | 0.44 | 0.33 | 0.39$\pm$0.02^**^ |
| DEX | 0.90 | 0.67 | 0.87 | 0.70 | 0.79$\pm$0.06^##^ |
| DEX/YOH | 0.46 | 0.49 | 0.63 | 0.48 | 0.520.09^$^ |
| YOH | 0.38 | 0.45 | 0.52 | 0.38 | 0.43$\pm$0.03 |
